# Triatomine Fauna and Recent Epidemiological Dynamics of Chagas Disease in an Endemic Area of Northeast Brazil

**DOI:** 10.1155/2018/7020541

**Published:** 2018-10-01

**Authors:** Cláudia M. Melo, Ana Carla F. G. Cruz, Antônio Fernando V. A. Lima, Luan R. Silva, Rubens R. Madi, Veronica de Lourdes S. Jeraldo, Ruben Mercado

**Affiliations:** ^1^Universidade Tiradentes, Programa de Pós-Graduação em Saúde e Ambiente, Laboratório de Doenças Infecciosas e Parasitárias (LDIP/ITP), Aracaju, Brazil; ^2^Secretaria de Estado da Saúde de Sergipe, Laboratório Central de Saúde Pública de Sergipe (Lacen-SE), Laboratório de Entomologia, Aracaju, Brazil; ^3^Universidade Tiradentes, Laboratório de Doenças Infecciosas e Parasitárias (LDIP/ITP), Aracaju, Brazil; ^4^Universidad de Chile, Facultad de Medicina, Santiago, Chile

## Abstract

Updated information of the dispersion dynamics of Chagas disease (CD) and a systemic analysis of these data will aid the early identification of areas that are vulnerable to transmission and enable efficient intervention. This work synthesized spatiotemporal information regarding triatomine fauna and analyzed this information in combination with the results from serological tests to elucidate the epidemiological panorama of CD in the state of Sergipe, Brazil. This is a retrospective analytical study that utilized information from the database of the National Chagas Disease Control Program. Between 2010 and 2016, 838 triatomines of eight species, namely, *Panstrongylus geniculatus,* which was first recorded in the state of Sergipe, *Panstrongylus lutzi, P. megistus, Triatoma brasiliensis, T. pseudomaculata, T. tibiamaculata, T. melanocephala*, and *Rhodnius neglectus,* were collected. Optical microscopy revealed that 13.2% of triatomines examined were infected by *Trypanosoma cruzi*-like flagellates. The distribution of triatomines exhibits an expanding south-central to northern dispersion, with a preference for semiarid and agreste areas and occasional observations in humid coastal areas due to anthropogenic actions reflected in the environment. Of the human cases analyzed from 2012 to 2016, 8.3% (191/2316) presented positive serology for *Trypanosoma cruzi*, and this proportion showed a gradual increase in the southern center of the state and new notifications in coastal regions. There is a need for intensification and continuity of the measures adopted by the Chagas Disease Control Program in Sergipe, identifying new priority areas for intervention and preferential ecotopes of the vectors, considering the occurrence of positive triatomines intradomicilliary and a source of new triatomines in the peridomiciles.

## 1. Introduction

Chagas disease (CD), which is a neglected disease caused by the protozoan *Trypanosoma cruzi*, represents one of the most significant public health problems in the world. Approximately 6-7 million people are estimated to be infected, particularly in Latin America, where CD is endemic [1, 2]. The estimates of Chagas disease show divergence in several countries due to different definitions of *Trypanosoma cruzi* infection, and as a result, the prevalence of CD in the Americas is difficult to determine. However, the number of infected individuals is very expressive and requires priority actions on the part of Latin American managers of basic healthcare [3].

The Brazilian Ministry of Health published the II Brazilian Consensus on Chagas Disease with the objective of systematizing strategies for the diagnosis, treatment, prevention, and control of Chagas disease in the country and to reflect on the available scientific evidence [4]. In Brazil, old and new health problems coexist with a predominance of chronic-degenerative diseases but with an expressive occurrence of transmissible diseases such as Chagas disease. This impacts the health of the northeast Brazilian population, in both cardiac and digestive clinical forms, and restricts access to diagnosis and therapy [5].

The modes of transmission of the disease with epidemiological relevance are the consumption of contaminated food, blood transfusion, congenital acquisition, organ transplantation, and laboratory and vector accidents [1]. The vector transmission of CD is mainly occurred by hematophagous triatomines of the genera *Panstrongylus*, *Rhodnius*, and *Triatoma*. These triatomines carry the infecting form of *Trypanosoma cruzi* in their fecal fluids, which can come in contact with the skin of vertebrate hosts such as humans and several mammals [6]. Sixty-four species of triatomines are currently registered in Brazil, and ten of them are of high epidemiological importance due to their behavioral characteristics [7–9].

Despite the advances in vector control and quality assurance of blood transfusions in several Latin American countries, particularly through intergovernmental initiatives [10–12], the relevance of Chagas disease as a public health problem remains unequivocal in Latin America, even though the epidemiological expression of CD exhibits different patterns and regional dynamics. In Brazil, the National Program for Vector Control of Chagas Disease reached its highest coverage in 1983, and a significant reduction of the triatomine-trypanosome indexes was achieved in the endemic area [13]. The National Health Foundation (*Fundação Nacional de Saúde*—FUNASA) has been supervising the (re)construction and/or remodeling of homes conducted by the Housing Improvement Program for the Control of Chagas Disease, which aims to improve the physical and sanitary conditions of homes and peridomicilies to avoid colonization of the insect vectors of Chagas disease. Twenty years later, and with the success of a set of measures adopted for the control of vector and transfusion transmissions, the Brazilian Ministry of Health received a certificate for the interruption of vector transmission of Chagas disease-causing *Triatoma infestans* from the Pan American Health Organization (PAHO). In addition to this indication of successful vector control, the last national survey of seroprevalence and evaluation of control of Chagas disease in Brazil indicated a drastic reduction of vector transmission in recent years [14]. However, acute cases of Chagas disease are still observed due to transmission by native species of triatomine bugs, which continue to invade and eventually colonize homes in different regions of Brazil [15–20] including the northeast region [21–23]. This situation leads to a reflection on the practical role of the certification of “interruption of the transmission of *Trypanosoma cruzi*” issued by PAHO [24].

The early identification of areas with greater (or significant) vulnerability to the occurrence of synanthropic triatomines and transmission of human Chagas disease is fundamental for the design and application of efficient and economically viable intervention actions [25]. The recognition of these areas can be aided by ecological studies, including ecological niche modeling techniques and from multicriteria analysis techniques coupled with geographic mapping studies and entomological, epidemiological, demographic, and environmental factors, to identify areas more suitable for the occurrence of triatomines [26]. There is no recent information regarding this health problem for the state of Sergipe, and thus, no systematic analysis has been conducted. The objective of this study was to synthesize (in a spatiotemporal manner) information regarding triatomine fauna and to analyze this information in combination with the results from serological tests to elucidate the epidemiological panorama of Chagas disease in the state of Sergipe.

## 2. Materials and Methods

This retrospective analytical study utilized information registered in the database of the National Chagas Disease Control Program (*Programa de Controle da Doença de Chagas*—PCDCh) in the state of Sergipe, northeast Brazil.

The northeast region of Brazil, which is one of the five geographical regions of the country, has an approximate population of 54 million inhabitants in an area of 1.3 million km^2^ and comprises nine states of the federation. This region is characterized by a small population density (41.48 inhabitants/km^2^), particularly in its rural areas. The northeast region is also one of the poorest regions of the country and remains very rural. In addition, this region presents the highest numbers of poor quality human dwellings, which are favorable for triatomine shelters.

**Figure 1:**
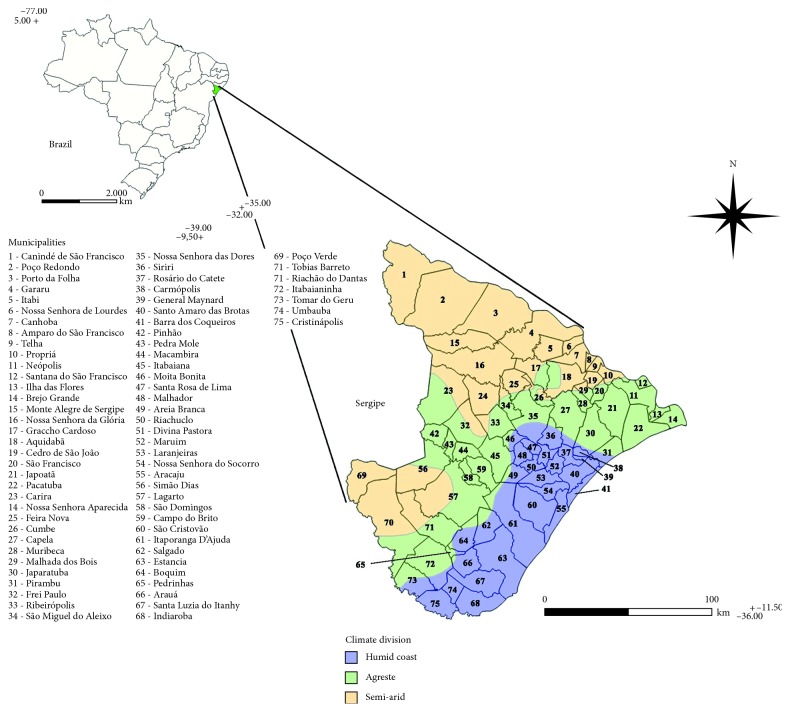
Location of the municipalities and climatic divisions in the state of Sergipe, Brazil. Source: SEMARH-Sergipe [28].

The state of Sergipe, which is one of the 27 federative units of Brazil, is situated in the northeast region of the country. It is the smallest of the Brazilian states and occupies a territorial area of 21,915 km^2^ with an estimated population of approximately 2.3 million inhabitants (population density = 94.36 inhabitants/km^2^) [27]. Its climate is characterized as tropical, which has alternating periods of drought (August to February, sometimes extending throughout the year in the interior of the state) and rains (more intense between March and July) and is more humid near the coast and drier in the interior. This distribution yields three climatic divisions: humid coast, agreste, and semiarid (Figure 1). In the state of Sergipe, the PCDCh is managed by the State Health Secretariat of Sergipe, Laboratory of Entomology of the Central Laboratory of Public Health of Sergipe (*Laboratório Central de Saúde Pública*—LACEN/SE), which serves all 75 municipalities in the state.

The historical series of the presence of triatomines in the municipalities of the state of Sergipe from 2010 to 2016 were analyzed according to the species, the positivity to trypanosomatids, and the geographical distribution. The triatomines from each municipality were collected by endemic agents through manual inspection and capture at residential sites. The inspections were conducted based on reports from residents or a previously scheduled active search.

The insects were collected using the selective method, that is, manual search with metallic tweezers and a flashlight to inspect openings and dark places, with a collection time of one hour/housing unit/individual. Pirisa® chemical dislodger was used to force insects to leave their resting places. Some specimens were received by spontaneous demand. The triatomines were stored in screw-cap flasks and sent to the Laboratory of Entomology of LACEN accompanied by their identification form. In the laboratory, the triatomines were identified at the species level using key of Galvão [6] and Carcavallo et al. [29].

The presence of flagellated trypanosomatids forms in fecal material was observed by removing the intestinal contents with scissors and forceps, macerated, and observed between slide and cover slip with one drop of buffered saline solution, using an optical microscope (400x magnification, running the entire cover slip). Two slides were prepared per examined specimen [30].

The distribution of triatomine occurrences by the municipality of the state of Sergipe was determined by the relative abundance of the triatomines found in each municipality and provides a dispersion trend for the triatomines in the territorial space of the state. The relative abundance (ra) is the percentage relation of the number of triatomines collected in the state (tt) with respect to the triatomines collected in the municipalities (tm) in the same period [ra(%) = (tm/tt) × 100] [31].

The Natural Infection Index was calculated to determine the potential risk of the municipality in relation to the transmission of Chagas disease. This index was calculated using the following equation:

(1) $$\begin{array}{c} \text{Natural Infection Index}\left(\text{NII}\right)=\frac{\text{number of infected triatomines }\times 100}{\text{number of examined triatomines}}. \end{array}$$

We also analyzed the records of the Laboratory Environment Manager system (*sistema Gerenciador de Ambiente Laboratorial*—GAL) with respect to the occurrence of Chagas disease in humans between 2012 (the year in which the GAL system was implemented) and 2016 assessed through an indirect immunofluorescence method using an IgG antibody.

Occurrence maps of triatomines and density (Kernel) were made representing the infestation and infection in the state (in the years 2010, 2012, and 2016), in addition to the percentages of positive serology for Chagas disease in the years 2012 and 2016, obtaining in this way the distribution in time and space. For the preparation of the maps, the free geoprocessing software Quantum GIS 2.18.15 (Free Software Foundation Inc.) was used.

The odds ratio was calculated for the presence of triatomines between the municipalities prioritized by the Housing Improvement Program and not prioritized. The Pearson correlation coefficient was also calculated relating the occurrence of triatomines and the Human Development Index (HDI) of municipalities. The tests were applied with the aid of the statistical software BioEstat 5.1 and adopting *α*=5%.

The study was conducted in accordance with the ethical norms detailed in Resolution 466 of 12/12/2012 of the National Health Council and approved by the Ethics Committee of the Tiradentes University (Protocol no. 070309).

## 3. Results and Discussion

**Figure 2:**
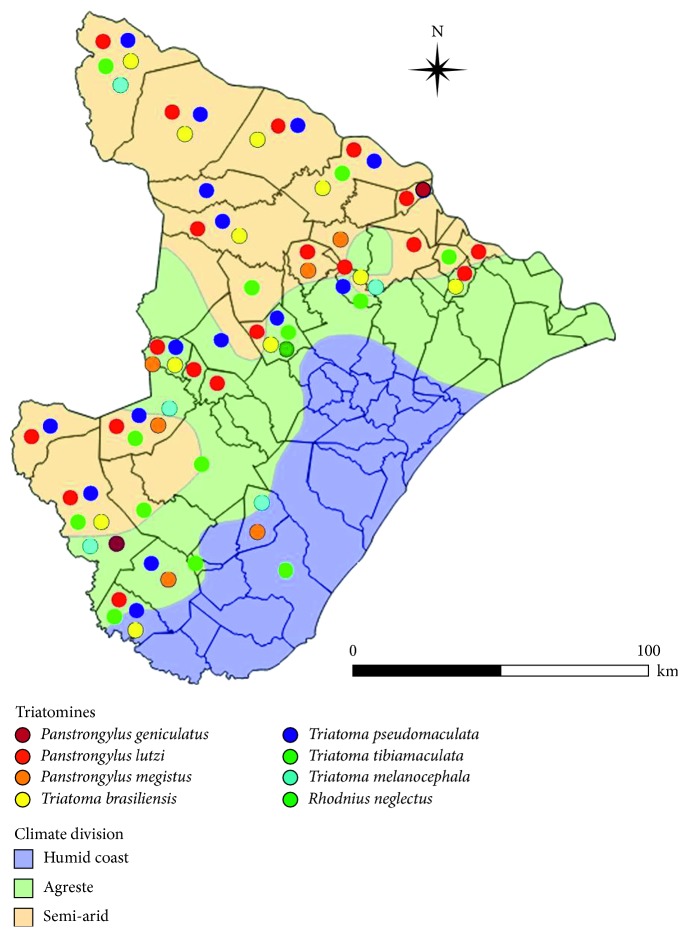
Distribution and climatic division of triatomines in the state of Sergipe, Brazil, from 2010 to 2016.

From 2010 to 2016, 838 triatomines of eight species, namely, *Panstrongylus geniculatus*, *P. lutzi*, *P. megistus*, *Triatoma brasiliensis*, *T. pseudomaculata*, *T. tibiamaculata*, *T. melanocephala*, and *Rhodnius neglectus* (Figure 2), were collected. Optical microscopy revealed that 13.2% of triatomines examined were infected by *Trypanosoma cruzi*-like flagellates. *Rhodnius neglectus* did not present positive results for *Trypanosoma cruzi*-like flagellate. It can be observed in Table 1 that the predominant species in the collections between 2014 and 2016 was *P. lutzi*, followed by *T. pseudomaculata* and *T. brasiliensis*. Most of the triatomines (87.9%) and those who were positive for *Trypanosoma cruzi*-like flagellates were collected in the intradomicile. Two nymphs of stage V were found in the inner house, while the others were in the peridomicile, it shows a tendency to the formation of colonies of triatomines in the home annexes.

All the collected species, which are considered vectors of CD, were previously described as occurring in the state of Sergipe by Dias et al. [32], with the exception of *P. geniculatus*, which was first observed in this study.

*Panstrongylus geniculatus* is a wild species that is particularly associated with armadillo burrows and limited vector competence of Chagas disease due to its low vector capacity [33]. Nevertheless, previous studies have detailed the colonization of home annexes in the state of Piauí [21] and the observation of positive specimens inside houses in Vitória de Santo Antão in the state of Pernambuco [34], and both of these states are located in the northeast region of Brazil.

Additionally, Fonseca et al. [35] found the same species described in this study in the interior of Rio Grande do Norte, which is located in the northeast region of Brazil, but none of their collected specimens presented positivity for *Trypanosoma cruzi*. Although all species of triatomines collected are potential vectors of this protozoan, there is not always a need to colonize the homes and/or peridomiciles in order to transmit human Chagas disease [24]. In this study, triatomines of the three genera were observed in domiciliary and peridomiciliary spaces with a high occurrence of four species of *Triatoma*, three species of *Panstrongylus*, and one species of *Rhodnius*.

From an epidemiological standpoint, the species *T. brasiliensis* and *T. pseudomaculata* are considered difficult to control in homes in the northeast region of Brazil. In this case, it is necessary in Sergipe continuous vigilance actions and with adequate responses to any evidence of constitution of intradomiciliary colonies [36].

The distribution of triatomines in the state of Sergipe appears to follow a climatic pattern with a higher concentration in semiarid and agreste areas. The latter, which are characterized as transition zone, are bounded to the east by the Atlantic forest and to the west by the caatinga. However, the lack of a rigid delimitation between climatic areas and because of the anthropic influence in the coastal region of the state has led to some observations of triatomines in municipalities within a humid climate area.

**Figure 3:**
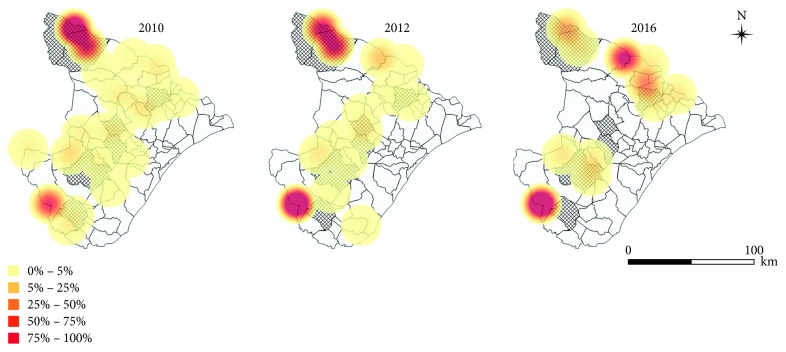
Spatial distribution (by density) of the percentage of infestation notification by triatomines in 2010, 2012, and 2016 in the state of Sergipe, Brazil. The hatching municipalities were considered at high risk for transmission of CD by the Sergipe State Health Department [37].

The distribution of the number of triatomine notifications throughout the state decreased during the analyzed period; however, a progressive tendency in the concentration of notifications in the northern region of the state, which is the area of the São Francisco River Basin, could be observed (Figure 3).

Between 2010 and 2016, thirty-two of the 75 municipalities in the state of Sergipe, that is, less than half, sent triatomine specimens for identification and analysis. In 2014, 60% of the triatomines captured in the state originated from the municipality of Tobias Barreto, followed by Canindé de São Francisco (8.7%), Porto da Folha (7.8%), and Gararu (6.8%).

According to the Chagas Disease Control Program of Sergipe, the following seven municipalities were classified as high risk for the transmission of the disease: Canindé de São Francisco, Poço Redondo, Aquidabã, Itabaiana, Itabaianinha, Ribeirópolis, and Lagarto [37]. However, as shown in Table 2, there was no record of a yearly triatomine encounter in some of these municipalities, even though they are classified as high-risk areas. The Tobias Barreto municipality, a medium-risk area located in the south-central region of the state, presented a higher infestation index than municipalities with a high risk of transmission, indicating a trend for a possible upward transition in the risk of vector transmission. This municipality borders an area classified by the Ministry of Health of Brazil as being at high risk for transmission (Itabaianinha).

Of the evaluated municipalities, 48.5% performed systematic triatomine collections and evaluations at least twice (in at least two different years) in the analyzed period, whereas 27.3% performed these evaluations more regularly (four to seven times). It is noteworthy that the current disarticulation and instability in the surveillance and control of triatomines, particularly in northeast region of Brazil, are due to the decentralization of Brazilian endemic control programs and the discontinuation of National Health Foundation (*Fundação Nacional de Saúde*—FUNASA) regional office, which complicate the structural and operational capacity of the municipalities with respect to the responsibility of the epidemiological surveillance of chagasic endemia [38] and the control of other endemic parasites (e.g., schistosomiasis and visceral leishmaniasis).

The epidemiological surveillance of the state of Sergipe has as a rule that encourages the population to notify the presence of suspected insects and to receive them through the Municipal Endemic Control. The routing of triatomines leads to a search (active search) in response to the notification and in the households where the triatomines were found; chemical control is carried out using insecticides of the pyrethroid class.

The Housing Improvement Program for the Control of Chagas Disease (*Programa de Melhorias Habitacionais para o Controle da Doença de Chagas*—MHCDCh) prioritizes locations in which dwellings favor the colonization of triatomines, and this classification is performed based on vulnerability to the vector transmission of CD and according to data from the Brazilian Ministry of Health [39]. The information on triatomines was registered in Sergipe and sent to the Health Surveillance Secretariat of the Ministry of Health of Brazil that subsidized the financing of approximately US$ 670,000 for the renovation and reconstruction of human dwellings in the eight eligible municipalities, and the benefits with respect to vector control should be reinforced by hygiene and (peri)domiciliary actions in the beneficiary communities.

The municipalities that were prioritized for the program are Aquidabã, Itabaiana, Itabaianinha, Lagarto, Macambira, Poço Redondo, Ribeirópolis, and Umbaúba. These municipalities began to receive benefits before the period included in this study, and the actions of the program were concluded in 2016. The housing improvements aimed to reduce the possibility of triatomine colonization in the home and peridomicile environments and showed a potential reduction in the occurrence of vectors positive for *Trypanosoma cruzi* close to the residents. For municipalities at potential risk for the transmission of CD, the profound environmental changes caused by agricultural production combined with the use of pesticides for controlling insects and other anthropogenic modifications should also be considered. In addition, the consequent reduction of native vegetation coverage and the preferential ecotype of the triatomines forced the vectors to move to the vicinity of human dwellings [22, 40]. As a result, the municipality of Cristinápolis, which is located in an area with only 9.64% of its original forest cover and eucalyptus production, was included even though no occurrence of vectors has been reported, but cases of positive serology for human CD were recorded [27, 41].

The municipalities that were not prioritized by the MHCDCh Program presented a 2.8-fold higher risk of triatomines observations than in the municipalities benefited by the Housing Improvement Program (OR = 2.842; CI = 0.873–9.644), which demonstrated that the building plastered brick houses and improvements outside the home reduce the possibility of triatomine detection.

The Human Development Index (HDI), which is calculated by the health, education, and income conditions of the population of the municipalities of the state of Sergipe, presented a negative and significant correlation with the presence of triatomines (*r*=−0.2768; *p*=0.017), that is, a lower HDI is associated with a greater chance of exposure to the vector in the home/peridomiciliary space. This fact demonstrates that a lack of knowledge regarding the transmission of Chagas disease due to unavailability of information coupled with poor housing conditions facilitates the colonization and the possibility of human infection by *Trypanosoma cruzi*.

Among the municipalities with evaluated triatomines, 15 presented positivity for *Trypanosoma cruzi*-like flagellates (Table 3). The municipalities of Tobias Barreto and São Francisco are of special interest because they are classified as areas of medium and low risk, respectively, but had higher rates of natural infection (higher than 20%). The municipality of São Francisco is located in the northeastern part of the state in an area considered by the Ministry of Health of Brazil as a low-risk area of transmission, and although analyses were not performed in all of the years covered by this study, high rates of natural infection demonstrate that the protozoan causing the CD tends to disperse to areas previously considered free of infection. This fact becomes more worrisome because this municipality is not located in a region that is historically transmitting Chagas disease, such as the center-south region of the state.

Natural infections may be underreported due to the low sensitivity of routine methodology employed by the official control organs (optical microscopy) [42], which indicates that extensive monitoring or more effective strategies are required to control the dispersion of the protozoan.

According to Dias and Coura [43], the dispersal of human Chagas disease depends on environmental factors related to anthropic actions, such as the migration of chagasic individuals from endemic regions and a significant alteration in the environmental scenario that permits invasion and eventual domiciliation of native species in newly constructed housing in rural areas. The economy of the municipality of São Francisco is based on agricultural production, silviculture, and the extraction of wood for use as firewood and charcoal, and only 2.7% of the natural forest in this municipality has been preserved [27], which might be contributing to the change in the epidemiological scenario of CD in the region.

**Figure 4:**
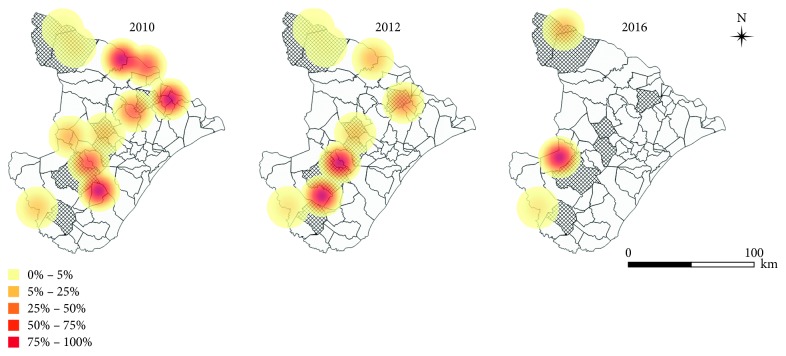
Geographic distribution (by density) of the percentage of cases of natural infection of triatomines by *Trypanosoma cruzi*-like flagellates in 2010, 2012, and 2016 in the state of Sergipe, Brazil. The hatching municipalities were considered at a high risk for transmission of CD by the Sergipe State Health Department [37].

This situation is also observed in other rural areas of the state and reflects an increase in the records of domiciled and peridomiciliary triatomines in historically nonendemic regions. This increase has altered the epidemiological dynamics of Chagas disease toward agreste and semiarid areas and increased the vulnerability of the population to chagasic infection (Figure 4).

Much of the interior of the state of Sergipe is characterized by small municipalities with a low population density (approximately 40 inhabitants/km^2^) and small rural properties intended for subsistence production [27]. Another common feature in the interior of this state is the construction of home annexes for housing animals (e.g., cattle, goats, pigs, and chickens). Lima et al. [22] found that the presence of positive triatomines in the rural properties of Sergipe is related to the variety of reared animals and the number of home annexes built to house these animals. Figueiredo et al. [44] observed a strong influence of ecological aggressions on the migration of triatomines toward man-made artificial ecotopes, because these places provide easy access to food, shelter, and protection against natural enemies.

The implementation of the GAL system by the Ministry of Health of Brazil permitted the centralization of information from the compulsory notification of diseases provided by the National System of Public Health Laboratories and supplied by epidemiological and analytical reports from regional networks of public health laboratories [45]. The GAL system was started in 2009 and was gradually deployed to the various Brazilian states (in Sergipe, it began to be implemented in 2012).

From the implementation of the GAL system in Sergipe to 2016, a total of 2,316 serological samples suspected of Chagas disease were analyzed in LACEN by indirect immunofluorescence for the detection of IgG antibodies to *Trypanosoma cruzi*, and 8.25% (191) of the samples presented positive serology for this protozoan. It is important to note that positive samples for the disease were observed in all the evaluated years (Table 4), which demonstrates the need for constant maintenance and active surveillance. Cross-reactivity with *Leishmania* sp. should also be considered because the state presents endemicity for visceral leishmaniasis [46, 47].

Population studies conducted in the south-central region of the state of Sergipe obtained varying prevalence rates for the adult population [48, 49], but these are still relevant and corroborated the seroprevalence indicated by the national serological survey of 1975/1980, which registered a 5.9% occurrence rate in the state of Sergipe [50]. It is important to note that this seroprevalence was obtained from a field study in which the Chagas disease-positive patients had a mean age of 47.2 years (27–63) [49].

Serological investigations for CD in children under seven years are fundamental as indicators of new cases of vector transmission. In the state of Sergipe, Teles et al. [51] did not find positivity in any of the 198 children examined from 1 to 14 years old, living in an area historically endemic to this parasite. Similarly, Ostermayer et al. [14] in a national serological survey conducted in children under five years of age, between 2001 and 2008, did not report positive cases of CD in the state of Sergipe.

In a serological survey conducted in the state of Piauí, in the northeast region of Brazil, Borges-Pereira et al. [52] found a prevalence of human CD ranging from 0.2 to 11.6% in 131 counties. A serological study in the state of Ceará, which is also located in the northeast region of Brazil, presented a global prevalence of 3.1%; however, the prevalence in villages in the rural area of the municipality ranged from 1.3 to 6.3% [53].

A relevant aspect with respect to the progression of CD is the existence of restrictive social policies and unequal production relations, which result in precarious housing in rural areas and easier access to food supplies and thus leading to the adaptation of triatomines to human dwellings. Thus, ecological, economic, and social determinants have made Chagas disease a public health problem, and for many years, this disease has been considered a “disease of poverty” [54–57].

Massaro et al. [58] evaluated the occurrence of CD in the municipality of Monte Negro in the state of Rondônia (northern region of Brazil) and found that 70.7% did not report a history of parasitic infection, but 3% of the population older than 50 years was infected. No child or young person showed positivity for the disease, and there was no natural infection in the region. A similar epidemiological situation was described by Diotaiuti et al. [59] in Ceará, where the rural population had an infection rate of ≤5%, and a higher prevalence was detected in the population over 50 years of age.

Sixty-four of the 75 municipalities in the state of Sergipe sent serum samples from suspected patients with CD in the evaluated period, and 22 were positive (Table 4). The discontinuity in sending the samples to accredited public health laboratories does not allow for a reliable understanding of Chagas disease in the state and makes it difficult to implement health policies that would aid in the control of parasitosis. The high prevalence rates found in the period for the state of Sergipe (Table 4) raise concerns regarding the municipalities that did not consistently adhere to the PCDCh, particularly those that were classified as a high risk for the transmission of CD such as Aquidabã (seven samples were sent in this period), Ribeirópolis (four samples), and Poço Redondo (two samples).

**Figure 5:**
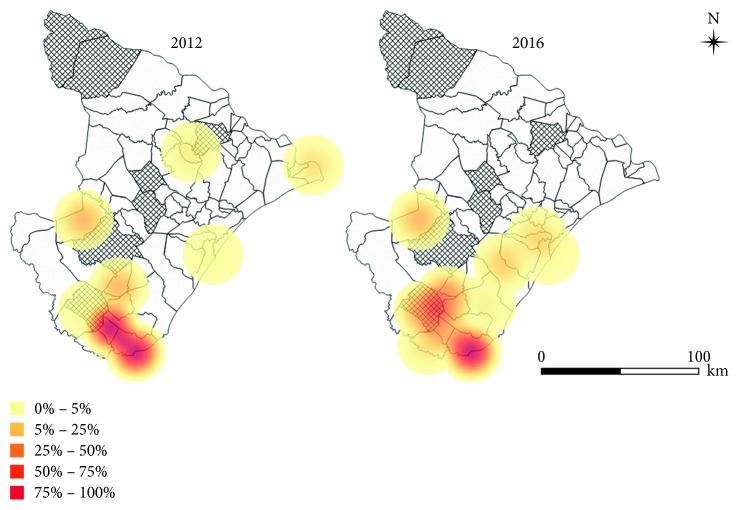
Geographic distribution (by density) of the percentage of serological reagents for *Trypanosoma cruzi* in human patients (indirect immunofluorescence—IgG) in 2012 and 2016 in the state of Sergipe, Brazil. The hatching municipalities were at high risk for the transmission of CD as described by the Sergipe State Health Department [37].

The municipalities located in the southern state of Sergipe revealed human cases with positive serology, which provide evidence of the presence of *Trypanosoma cruzi* in region, and higher concentrations of cases were recorded in the municipalities of Umbaúba, Cristinápolis, Tomar do Geru, and Itabaianinha (Figure 5). Through a field study of triatomines, Lima et al. [22] found that the Natural Infection Index for the infection of *Panstrongylus megistus* species by *Trypanosoma cruzi*-like flagellates was 31.1%. These samples were collected in the intra- and peridomiciles of rural dwellings in the municipality of Itabaianinha. It is important to emphasize that actions for the control of CD should be strengthened in this region because entomological collection has been recently performed intermittently and/or not performed in these municipalities in recent years.

Even though CD is considered a controlled disease, local revitalization of the Chagas Disease Control Program is necessary because this disease was observed in rural and periurban areas of Sergipe municipalities that were previously not considered at risk. Instead of neglect or discontinuity, this work supports timely action and optimization of human and structural resources for the surveillance and prevention of CD, particularly in the context of joint detection of human cases, intensification of anthropogenic actions, demographic growth, and the detection of various species of vectors and mammals with potential infection.

The need for both early diagnosis and adequate treatment of diagnosed patients (chronic cases) is predominant in a region in which the population is neglected due to their various difficulties in accessing basic healthcare, such as in the rural area of the southern center of Sergipe.

## 4. Conclusions

Our results show that the epidemiological panorama of Chagas disease in the state of Sergipe reveals a gradual increase of seroprevalence and is concentrated in the south-central part of the state with new notifications in the coastal region. The dispersion of domiciled triatomines (vectors) extends in the south-central direction to the north. Of the eight triatomines species registered in Sergipe during the period evaluated, seven were positive for *Trypanosoma cruzi*-like flagellates, and therefore, the risk of CD transmission at the notification sites of this insect could not be neglected. Thus, there is a need for systematic entomological and seroepidemiological surveys to understand the dynamics and risk of autochthonous transmission, especially in young populations, in rural and periurban areas, in the northern and coastal regions of the state of Sergipe.

Thus, it is important to intensify the actions of Chagas Disease Control Program in Sergipe focused on the identification and intervention in new areas at risk of human transmission, considering the triatomine notifications in intra- or peridomicile and environmental conditions.

This first record of *Panstrongylus geniculatus* in the peridomiciliary area of Sergipe also serves as an alert regarding the transmission potential of the species, and its evolution should be monitored by entomological surveillance.

## Figures and Tables

**Table 1:** Triatomines collected between the years 2014 and 2016^*∗*^ in the state of Sergipe.

Species^*∗∗*^	Negative							Positive		
	Nymphs				Female	Male	NI	Female	Male	Total
	II	III	IV	V						
*P. geniculatus*	—	—	—	—	—	1	—	—	—	1
*P. lutzi*	—	—	—	—	7	67	—	2	12	88
*P. megistus*	—	—	—	—	—	1	—	—	—	1
*T. brasiliensis*	1	4	4	5	11	14	—	—	—	37
*T. melanocephala*	—	—	1	—	—	2	—	—	1	
*T. pseudomaculata*	—	—	—	—	24	51	2	—	4	81
*T. tibiamaculata*	—	—	—	—	5	4	—	1	—	10
Others^*∗∗∗*^										51

^*∗*^Considering only the period between 2014 and 2016 for the use of a systematized methodology for counting and identification (taxonomy and stage of development) of triatomines collected during the phase of consolidation of entomological surveillance of the National Chagas Disease Control Program from that adopted between 2010 and 2013. ^*∗∗*^The species *Rhodnius neglectus* was recorded between the years 2010 and 2013. ^*∗∗∗*^Nonhematophagous triatomines. NI: not identified.

**Table 2:** Relative abundance of triatomines from 2010 to 2016 in the state of Sergipe, Brazil.

Risk^*∗*^	Municipality	2010	2011	2012	2013	2014	2015	2016	Average period
High	Aquidabã	1.04	0.00	1.35	1.94	4.85	1.28	2.78	1.89
	Canindé São Francisco	36.46	10.71	22.30	33.98	8.74	19.23	8.33	19.96
	Itabaianinha	0.52	0.00	0.00	0.00	0.00	0.00	0.00	0.07
	Lagarto	0.00	0.00	0.00	0.00	0.00	0.00	2.78	0.40
	Poço Redondo	19.79	34.29	20.95	9.71	0.00	0.00	2.78	12.50
	Ribeirópolis	2.08	0.00	2.70	24.27	2.91	2.56	0.00	4.93

Medium	Cedro de São João	0.52	0.00	0.00	0.00	0.00	0.00	0.00	0.07
	Cumbe	2.60	0.00	0.00	0.00	1.94	1.28	0.00	0.83
	Estancia	0.00	0.00	0.00	0.00	0.00	1.28	0.00	0.18
	Feira Nova	1.04	0.00	0.00	0.00	1.94	0.00	0.00	0.43
	Frei Paulo	0.52	0.00	0.00	0.00	0.00	0.00	0.00	0.07
	Gararu	1.56	0.00	0.68	2.91	6.80	14.10	2.78	4.12
	Gracho Cardoso	0.00	0.00	0.68	0.00	0.00	0.00	0.00	0.10
	Itabi	0.00	0.00	0.00	0.00	0.00	0.00	11.11	1.59
	Macambira	0.00	0.00	0.00	0.00	0.00	1.28	0.00	0.18
	Monte Alegre de Sergipe	0.52	0.00	0.00	0.00	0.00	0.00	0.00	0.07
	Nossa Senhora Aparecida	0.00	0.00	1.35	0.00	0.00	0.00	0.00	0.19
	Nossa Senhora da Gloria	1.56	0.00	0.00	6.80	0.00	0.00	0.00	1.19
	Nossa Senhora de Lourdes	0.52	0.00	0.00	0.00	0.97	1.28	0.00	0.40
	Pedra Mole	0.00	0.00	0.68	0.00	0.00	0.00	0.00	0.10
	Pedrinhas	0.00	0.71	0.00	0.00	0.00	0.00	0.00	0.10
	Poço Verde	1.56	0.00	0.00	1.94	0.00	2.56	0.00	0.87
	Porto da Folha	0.52	0.00	6.08	3.88	7.77	15.38	22.22	7.98
	Riachão do Dantas	0.00	0.00	0.68	0.00	0.00	0.00	0.00	0.10
	Salgado	0.52	0.00	0.00	0.00	0.00	0.00	0.00	0.07
	Santa Luzia do Itanhy	0.00	0.00	0.68	0.00	0.00	0.00	0.00	0.10
	Simão Dias	4.17	22.14	2.70	0.00	0.97	0.00	2.78	4.68
	Tobias Barreto	18.23	26.43	38.51	14.56	60.19	38.46	38.89	33.61
	Tomar do Geru	2.08	2.14	0.00	0.00	2.91	0.00	0.00	1.02

Low	Propriá	0.00	0.00	0.00	0.00	0.00	0.00	2.78	0.40
	São Francisco	1.56	2.14	0.68	0.00	0.00	0.00	2.78	1.02

UC	Pinhão	1.56	1.43	0.00	0.00	0.00	1.28	0.00	0.61

Total municipalities evaluated per year		21	8	14	9	11	12	11	—

^*∗*^Risk of transmission of CD according to the classification guidelines of the Ministry of Health. UC: unclassified [37].

**Table 3:** Natural Infection Index (%) of triatomines by *Trypanosoma cruzi*-like flagellates from 2010 to 2016 in the state of Sergipe, Brazil.

Risk^*∗*^	Municipality	2010	2011	2012	2013	2014	2015	2016	Average period
High	Aquidabã	0.00	—	50.00	0.00	20.00	0.00	0.00	10.00
	Canindé São Francisco	8.57	20.00	6.06	2.86	0.00	0.00	33.33	10.12
	Poço Redondo	10.53	6.25	6.45	10.00	—	—	0.00	4.75

Medium	Cedro de São João	100.00	—	—	—	—	—	—	14.29
	Cumbe	60.00	—	—	—	0.00	—	—	8.57
	Gararu	66.67	—	0.00	0.00	28.57	0.00	0.00	13.61
	Nossa Senhora da Gloria	0.00	—	—	28.57	—	—	—	4.08
	Porto da Folha	100.00	—	33.33	0.00	0.00	0.00	0.00	19.05
	Riachão do Dantas	—	—	100.00	—	—	—	—	14.29
	Ribeirópolis	25.00	—	25.00	0.00	0.00	0.00	—	7.14
	Salgado	100.00	—	—	—	—	—	—	14.29
	Simão Dias	0.00	9.68	0.00	—	0.00	—	100.00	15.67
	Tobias Barreto	25.71	45.95	15.79	40.00	4.84	26.67	14.29	24.75

Low	São Francisco	66.67	33.33	100.00	—	—	—	0.00	28.57

UC	Pinhão	33.33	100.00	—	—	—	0.00	—	19.05

^*∗*^Risk of transmission of CD according to the classification guidelines of the Ministry of Health. UC: unclassified [37].

**Table 4:** Percentage of positive serology for Chagas disease in human patients based on the detection of IgG antibodies against *Trypanosoma cruzi* by indirect immunofluorescence and according to municipalities of the state of Sergipe, Brazil, from 2012 to 2016.

Risk^*∗*^	Municipality	2012	2013	2014	2015	2016	Average period
High	Itabaiana	—	0.00	0.00	20.00	0.00	4.00
	Itabaianinha	6.67	50.00	13.04	46.15	50.00	33.17
	Lagarto	0.00	0.00	8.00	0.00	0.00	1.60

Medium	Arauá	0.00	0.00	—	33.33	—	6.67
	Boquim	33.33	0.00	0.00	7.14	0.00	8.10
	Cristinápolis	0.00	30.00	71.43	11.11	17.65	26.04
	Cumbe	16.67	—	—	—	—	3.33
	Estancia	11.54	8.79	10.96	1.96	9.09	8.47
	Indiaroba	100.00	100.00	0.00	0.00	100.00	60.00
	Pedrinhas	—	14.29	0.00	0.00	50.00	12.86
	Simão Dias	33.33	50.00	33.33	20.00	33.33	34.00
	Tobias Barreto	0.00	0.00	0.75	13.33	0.00	2.82
	Tomar do Geru	—	33.33	—	—	—	6.67
	Umbauba	100.00	5.26	19.23	9.57	37.14	34.24

Low	Itaporanga D'Ajuda	0.00	16.67	33.33	0.00	25.00	15.00

No risk	Brejo Grande	—	100.00	—	0.00	0.00	20.00
	Carmópolis	0.00	100.00	0.00	—	—	20.00
	Japoatã	—	50.00	—	—	0.00	10.00
	Nossa Senhora do Socorro	0.00	0.00	15.38	11.11	20.00	9.30
	São Cristóvão	0.00	0.00	0.00	16.67	0.00	3.33
	Aracaju	4.76	8.47	6.25	14.81	5.71	8.00

UC	Pinhão	—	25.00	0.00	—	—	5.00

	Total prevalence/year	3.60	9.19	5.56	9.74	17.62	

^*∗*^Risk for the transmission of CD according to the classification guidelines of the Ministry of Health. UC: unclassified [37]. Source: Central Laboratory of Public Health of Sergipe (LACEN/SE).

## Data Availability

The data that support the results of this study can be made available by official request to the corresponding author (C.M.M.). Some data are not publicly available because they contain information that may compromise the privacy of research participants. The following data related to the article can be accessed: 1. Situational report of Chagas disease in the state of Sergipe available at http://bvsms.saude.gov.br/publicacoeshttp://www.funasa.gov.br/melhorias-habitacionais-para-o-controle-da-doenca-de-chagas—some data are not in the public domain, since they identify the residence and its respective resident, and according to the ethical standards contained in Resolution 466 of 12/12/2012 of the National Health Council of Brazil, the secrecy and confidentiality of patients' identity and information must be guaranteed. 2. Data from the Laboratory Environment Manager system (GAL—sistema Gerenciador de Ambiente Laboratorial) available at http://gal.sergipe.sus.gov.br—with access to the data by confidentiality agreement, in accordance with the ethical standards contained in Resolution 466 of 12/12/2012 of the National Health Council of Brazil, which guarantees the privacy of the identity and secrecy of patient information. 3. Municipalities' information available on the public website of the Brazilian Institute of Geography and Statistics (IBGE—Instituto Brasileiro de Geografia e Estatística) (https://cidades.ibge.gov.br/)
